# Molecular Mechanism of Stem Cell Differentiation into Adipocytes and Adipocyte Differentiation of Malignant Tumor

**DOI:** 10.1155/2020/8892300

**Published:** 2020-08-12

**Authors:** Kexin Zhang, Xudong Yang, Qi Zhao, Zugui Li, Fangmei Fu, Hao Zhang, Minying Zheng, Shiwu Zhang

**Affiliations:** ^1^Department of Pathology, Tianjin Union Medical Center, Tianjin, China; ^2^Nankai University School of Medicine, Nankai University, Tianjin, China; ^3^Tianjin Rehabilitation Center, Tianjin, China; ^4^Graduate School, Tianjin University of Traditional Chinese Medicine, Tianjin, China

## Abstract

Adipogenesis is the process through which preadipocytes differentiate into adipocytes. During this process, the preadipocytes cease to proliferate, begin to accumulate lipid droplets, and develop morphologic and biochemical characteristics of mature adipocytes. Mesenchymal stem cells (MSCs) are a type of adult stem cells known for their high plasticity and capacity to generate mesodermal and nonmesodermal tissues. Many mature cell types can be generated from MSCs, including adipocyte, osteocyte, and chondrocyte. The differentiation of stem cells into multiple mature phenotypes is at the basis for tissue regeneration and repair. Cancer stem cells (CSCs) play a very important role in tumor development and have the potential to differentiate into multiple cell lineages. Accumulating evidence has shown that cancer cells can be induced to differentiate into various benign cells, such as adipocytes, fibrocytes, osteoblast, by a variety of small molecular compounds, which may provide new strategies for cancer treatment. Recent studies have reported that tumor cells undergoing epithelial-to-mesenchymal transition can be induced to differentiate into adipocytes. In this review, molecular mechanisms, signal pathways, and the roles of various biological processes in adipose differentiation are summarized. Understanding the molecular mechanism of adipogenesis and adipose differentiation of cancer cells may contribute to cancer treatments that involve inducing differentiation into benign cells.

## 1. Introduction

Adipogenesis is the process through which mesenchymal stem cells (MSCs) commit to the adipose lineage and differentiate into adipocytes. During this process, preadipocytes cease to proliferate, begin to accumulate lipid droplets, and develop morphologic and biochemical characteristics of mature adipocytes, such as hormone-responsive lipogenesis and lipolytic programs. Currently, there are mainly two models of benign adipocyte differentiation *in vitro*. One is fibroid pluripotent stem cells, which can differentiate into not only adipocytes, but also muscle, cartilage, and other cells. There are two kinds of fibroid pluripotent stem cells: bone marrow and adipose mesenchymal stem cells. Another group is fibroblastic preadipocytes, which have a single direction of differentiation, namely, lipid differentiation, including 3T3-L1, and 3T3-F422A cells [1]. Cancer cells with tumor initiation ability, designated as cancer stem cells (CSCs), have the characteristics of tumorigenesis and the expression of specific stem cell markers, as well as the long-term self-renewal, proliferation capacity, and adipose differentiation potential [2]. In addition to CSCs [2], cancer cells undergoing epithelial-mesenchymal transformation (EMT) have been reported to be induced to differentiate into adipocytes [3–5]. Lung cancer NCI-H446 cells can be induced to differentiate into neurons, adipocytes, and bone cells in vitro [6]. The adipogenesis differentiation treatment is promising in the *p*53 gene deletion type of fibroblast-derived cancer [7]. Cancer cells with homologous recombination defects, such as ovarian and breast cancer cells with breast cancer susceptibility genes (BRCA) 1/2 mutations, can be induced to differentiate by poly ADP-ribose polymerase (PARP) inhibitors [2]. The nuclear receptor peroxisome proliferator-activated receptor *γ* (PPAR*γ*) agonist (antidiabetic, thiazolidinedione drug) can induce growth arrest and adipogenic differentiation in human, mouse, and dog osteosarcoma cells [8]. Thyroid cancer cells expressing the PPAR*γ* fusion protein (PPFP) can be induced to differentiate into adipocytes by pioglitazone [9]. Adipogenesis can be induced in well-differentiated liposarcoma (WDLPS) and dedifferentiated liposarcoma (DDLPS) cells by dexamethasone, indomethacin, insulin, and 3-isobutyl-1-methyl xanthine (IBMX) [10].

In this review, we highlight some of the crucial transcription factors that induce adipogenesis both in MSCs and in CSCs, including the well-studied PPAR*γ* and CCAAT enhancer-binding proteins (C/EBPs) [11], as well as other cell factors that have been recently shown to have an important role in adipocyte differentiation. We focus on understanding the complex regulatory mechanism of adipocyte differentiation that can contribute to the clinical treatment of human diseases, including those caused by obesity and adipocytes dysfunction, especially for the malignant tumor, which can be transdifferentiated into mature adipocytes.

## 2. Adipocyte Differentiation

Cell proliferation and differentiation are two opposing processes, and there is a transition between these two processes in the early stages of adipocyte differentiation. The interaction of cell cycle regulators and differentiation factors produces a cascade of events which ultimately results in the expression of adipocyte phenotype [7]. Adipogenesis has different stages. Each stage has a specific gene expression pattern [12]. In general, adipocyte differentiation of pluripotent stem cells is divided into two phases. The first phase, known as determination, involves the commitment of pluripotent stem cells to preadipocytes. The preadipocytes cannot be distinguished morphologically from their precursor cells, but also have lost the potential to differentiate into other cell types. In the second phase, which is known as terminal differentiation, the preadipocytes gradually acquire the characteristics of mature adipocytes and acquire physiological functions, including lipid transport and synthesis, insulin sensitivity, and the secretion of adipocyte-specific proteins [13].

The differentiation of precursor adipocytes is also divided into four stages: proliferation, mitotic cloning, early differentiation, and terminal differentiation [14]. After the precursors are inoculated into the cell culture plates, the cells grow exponentially until they converge. After reaching contact inhibition, the growth rate slows and gradually stagnates, and the proliferation of precursor adipocytes stops, which is very necessary for initiating the differentiation of precursor adipocytes. Adipocyte precursors exhibit transient mitosis, called “clonal expansion,” a process that relies on the action of induced differentiation factors. Some preadipocyte cells (mouse cell lines 3T3-L1, 3T3-F442A) undergo one or two rounds of cell division prior to differentiation [15], whereas other cell lines (mouse C3H10T1/2) differentiating into adipocyte do not undergo mitosis clonal expansion [16]. Whether “mitotic clonal expansion” is required for adipose differentiation remains controversial. However, it is certain that some of the checkpoint proteins for mitosis regulate aspects of adipogenesis [7, 17]. When cells enter the terminal differentiation stage, the *de novo* synthesis of fatty acids increases significantly, the transcription factors and adipocyte-related genes work cooperatively to maintain precursor adipocyte differentiation into mature adipocytes containing large lipid droplets [1].

## 3. Regulatory Pathways in Preadipocytes Commitment

**Figure 1:**
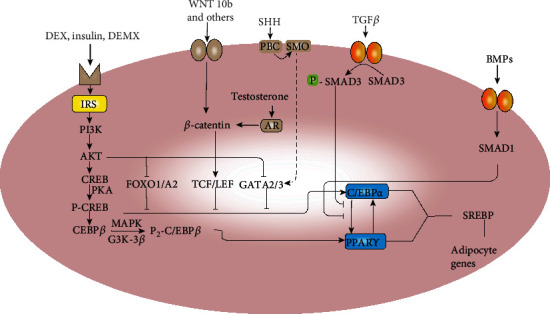
Regulation pathways in preadipocytes commitment. BMP and Wnt families are mediators of MSCs commitment to produce preadipocytes. Exposure of growth-arrested preadipocytes to differentiation inducers (IGF1, glucocorticoid, and cAMP) triggers DNA replication, leading to adipocyte gene expression due to a transcription factor cascade. The dotted line indicates an uncertain molecular regulatory mechanism.

Adipocyte differentiation is a complex process in which gene expression is finely regulated. The most basic regulatory network of adipose differentiation has not been updated in recent years, but some factors and signaling pathways that do affect adipose differentiation have been continuously reported. Adipocyte differentiation is the result of the gene expression that determines the phenotype of adipocytes, which is a complex and delicate regulatory process (Figure 1).

### 3.1. Wnt Signal Pathway in Adipogenesis

Wnt signaling is important for adipocytes proliferation and differentiation both *in vitro* and *in vivo* [18]. The Wnt family of secreted glycoproteins functions through paracrine and autocrine mechanisms to influence cell fate and development. Wnt protein binding to frizzled receptors initiates signaling through *β*-catenin-dependent and -independent pathways [19]. Wnt signaling inhibits adipocyte differentiation in vitro by blocking the expression of PPAR*γ* and C/EBP*α* [20]. Constitutive Wnt10b expression inhibits adipogenesis. Wnt10b is expressed in preadipocytes and stromal vascular cells, but not in adipocytes. In vivo, transgenic expression of Wnt10b in adipocytes results in a 50% reduction in white adipose tissue mass and absent brown adipose tissue development [21]. Wnt10a and Wnt6 have also been identified as determinants of brown adipocyte development [22, 23]. Wnt5b is transiently induced during adipogenesis and promotes differentiation [24], indicating that preadipocytes integrate inputs from several competing Wnt signals.

### 3.2. The Hedgehog (HH) Signaling Pathway Mechanism

Three vertebrate HH ligands including sonic hedgehog (SHH), Indian hedgehog (IHH), and desert hedgehog (DHH) have been identified and initiated a signaling cascade mediated by patched (Ptch-1 and Ptch-2) receptors [25, 26]. HH signaling had an inhibitory effect on adipogenesis in murine cells, such as C3H10T1/2, KS483, calvaria MSCs lines, and mouse adipose-derived stromal cells [27]. These cells were visualized by decreased cytoplasmic fat accumulation and the expression of adipocyte marker genes after HH signaling was inhibited [28]. Although it is generally agreed that HH expression has an inhibitory effect on preadipocyte differentiation, the mechanisms linking HH signaling and adipogenesis remain poorly defined [29].

### 3.3. ERK/MAPK/PPAR Signal Pathway

Extracellular-regulated protein kinase (ERK) is required in the proliferative phase of differentiation. ERK activity blockade in 3T3-L1 cells and embryonic stem cells can inhibit adipogenesis. In the terminal differentiation phase, ERK1 activity leads to PPAR*γ* phosphorylation, which inhibits adipocyte differentiation. This implies that ERK1 activity must be reduced after adipocyte proliferation so that differentiation can proceed. This reduction is mediated in part by mitogen-activated protein kinase (MAPK) phosphatase-1 (MKP1) [30, 31]. These extracellular and intracellular regulation factors cause adipocyte-specific gene expression and eventually lead to adipocyte formation.

## 4. Adipocyte Differentiation Regulatory Proteins

### 4.1. PPAR*γ* and Adipocyte Differentiation

PPAR*γ* is a member of the nuclear-receptor superfamily and is both necessary and sufficient for adipogenesis [32]. Forced expression of PPAR*γ* is sufficient to induce adipocyte differentiation in fibroblasts [33]. Indeed, the proadipogenic C/EBPs and Krüppel-like factors (KLFs) have all been shown to induce at least one of the two PPAR*γ* promoters. In contrast, antiadipogenic transcription factor GATA functioned in part by repressing PPAR*γ* expression [34]. PPAR*γ* itself has two isomers. The relative roles of PPAR*γ*1 and PPAR*γ*2 in adipogenesis remain an open question. PPAR*γ*2 is mainly expressed in adipose tissue, while PPAR*γ*1 is expressed in many other tissues. Although both can promote adipocyte differentiation, PPAR*γ*2 could do so effectively at very low ligand concentration compared with PPAR*γ*1 [35]. The two protein isoforms are generated by alternative splicing and promoter usage, and both are induced during adipogenesis. PPAR*γ*1 can also be expressed in cell types other than adipocytes. Ren et al. [36] used engineered zinc-finger proteins to inhibit the expression of the endogenous PPAR*γ*1 and PPAR*γ*2 promoters in 3T3-L1 cells. Ectopic expression of PPAR*γ*2 promotes adipogenesis, whereas that of PPAR*γ*1 does not. Zhang et al. reported that PPAR*γ*2 deficiency impairs the development of adipose tissue and insulin sensitivity [37].

There are transcriptional cascades between adipocytes genes, including PPAR*γ* and C/EBP*α* which are the core adipocyte differentiation regulators. In the early stage of adipocyte differentiation, the expression of C/EBP*β* and C/EBP*δ* increase, which upregulates C/EBP*α* expression, further activate PPAR*γ*. PPAR*γ* activating C/EBP*α* in turn results in a positive feedback. PPAR*γ* binding with retinoic acid X receptor (RXR) forms different heterodimers. The various dimmers can combine with the PPAR*γ* response element (PPRE) and initiate the transcription of downstream genes for differentiation into adipocytes [38].

**Figure 2:**
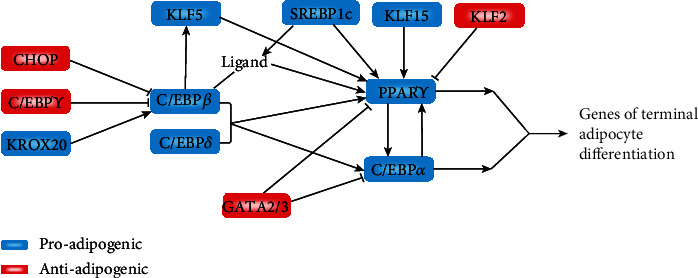
A cascade of transcription factors that regulate adipogenesis. PPAR*γ* is one of the key transcription factors in adipogenesis and the core of the transcriptional cascade that regulates adipogenesis. PPAR*γ* expression is regulated by several proadipogenic (blue) and antiadipogenic (red) factors. C/EBP*α* is regulated through a series of inhibitory protein–protein interactions. Some transcription factor families include several members that participate in adipogenesis, such as the KLFs. Black lines indicate effects on gene expression; violet lines represent effects on protein activity.

C/EBPs participate in adipogenesis, and several C/EBP family members are expressed in adipocytes, including C/EBP*α*, C/EBP*β*, C/EBP*γ*, C/EBP*δ*, and C/EBP-homologous protein (CHOP). The temporal expression of these factors during adipocyte differentiation triggers a cascade whereby early induction of C/EBP*β* and C/EBP*δ* leads to C/EBP*α* expression. This notion is further supported by the sequential binding of these transcription factors to several adipocyte promoters during adipocyte differentiation. C/EBP*β* is crucial for adipogenesis in immortalized preadipocyte lines. C/EBP*β* and C/EBP*δ* promote adipogenesis at least in part by inducing C/EBP*α* and PPAR*γ*. C/EBP*α* induces many adipocyte genes directly and plays an important role in adipose tissue development. Once C/EBP*α* is expressed, its expression is maintained through autoactivation [39]. Despite the importance of C/EBPs in adipogenesis, these transcription factors clearly cannot function efficiently in the absence of PPAR*γ*. C/EBP*β* cannot induce C/EBP*α* expression in the absence of PPAR*γ*, which is required to release histone deacetylase-1 (HDAC1) from the C/EBP*α* promoter [40]. Furthermore, ectopic C/EBP*α* expression cannot induce adipogenesis in PPAR*γ*^–/–^ fibroblasts [41]. However, C/EBP*α* also plays an important role in differentiated adipocytes. Overexpression of exogenous PPAR*γ* in C/EBP*α*-deficient cells showed that, although C/EBP*α* is not required for lipid accumulation and the expression of many adipocyte genes, it is necessary for the acquisition of insulin sensitivity [42, 43] (Figure 2). Human fibroblasts with the ability to differentiate into adipocytes also do not undergo mitotic cloning amplification. However, PPAR*γ* exogenous ligands need to be added to promote adipocyte differentiation. Therefore, it can be inferred that mitotic cloning expansion can produce endogenous ligands of PPAR*γ* [7].

### 4.2. BMP and Transforming Growth Factor *β* (TGF-*β*) in Adipocyte Differentiation

A variety of extracellular factors affect the preadipocyte commitment of stem cells, including bone morphogenetic protein (BMP) [44], transforming growth factor *β* (TGF-*β*) [45], insulin/insulin-like growth factor 1 (IGF1) [46], tumor necrosis factor *α* and interleukin 1 *β* [47], matrix metalloproteinase 2 [48], fibroblast growth factor (FGF) 1, and FGF2 [49]. BMP and TGF-*β* have varied effects on the differentiation fate of mesenchymal cells [50]. The TGF-*β* superfamily members, BMPs, and myostatin regulate the differentiation of many cell types, including adipocytes [51]. TGF-*β* inhibitor can promote adipose differentiation of cancer cells with a mesenchymal phenotype in vitro, and transgenic overexpression of TGF-*β* impairs adipocyte development [3]. Inhibition of adipogenesis could be obtained through blocking of endogenous TGF-*β* with a dominant-negative TGF-*β* receptor or drosophila mothers against decapentaplegic protein (SMAD) 3 inhibition. SMAD3 binds to C/EBPs and inhibits their transcriptional activity, including their ability to transactivate the PPAR*γ*2 promoter [52, 53]. Exposure of multipotent mesenchymal cells to BMP4 commits these cells to the adipocyte lineage, allowing them to undergo adipose conversion [50]. The effects of BMP2 are more complex and depend on the presence of other signaling molecules. BMP2 alone has little effect on adipogenesis, and it interacts with other factors such as TGF-*β* and insulin to stimulate adipogenesis of embryonic stem cells [54]. BMP2 stimulates adipogenesis of multipotent C3H10T1/2 cells at low concentrations and can contribute to chondrocyte and osteoblast development at higher concentrations [55].

### 4.3. KLFs in Adipocyte Differentiation

During adipocyte differentiation, some KLF family members are overexpressed, such as KLF4, KLF5, KLF9, and KLF15, while KLF16 expression is reduced [56, 57]. KLF15 is the first KLF family members, which were identified to be involved in adipocyte differentiation. Its expression increased significantly on the sixth day of 3T3-L1 adipocyte differentiation and peaked on the second day of adipocyte induction in MSCs and mouse embryonic fibroblasts. Inhibition of KLF15 by siRNA or mutation led to a decrease in PPAR*γ*, CEBP*α*, fatty acid-binding protein 4 (FABP4), and glucose transporter 4 (GLUT4). However, overexpression of KLF15 in NIH3T3 cells was found to be associated with lipid accumulation as well as increases in PPAR*γ* and FABP4 [58]. Mice with complete absence of KLF5 showed embryonal lethality, and mice with single-chromosome KLF5 knockout showed a significant reduction in white fat in adulthood, suggesting that KLF5 plays an important role in adipocyte differentiation. KLF5 can be activated by C/EBP*β* or C/EBP*δ*, which is involved in early adipocyte differentiation. KLF5 can be activated by C/EBP*β* or C/EBP*δ*, which is involved in early adipocyte differentiation. Direct binding of KLF5 to the PPAR*γ*2 promoter in combination with C/EBPs induces PPAR*γ*2 expression [59]. Transfection of KLF5 dominant-negative mutants in 3T3-L1 cells reduced lipid droplet accumulation and inhibited PPAR*γ* and C/EBP*α* expression, whereas overexpression of wild KLF5 significantly promoted adipocyte differentiation, even without exogenous hormone stimulation. Similar to KLF5, KLF9 knockdown can inhibit the expression of a series of adipocyte differentiation genes, such as PPAR*γ*, C/EBP*α*, and FABP4, hence inhibiting adipocyte differentiation. However, KLF9 overexpression did not upregulate the expression of PPAR*γ* and C/EBP*α* [60]. In addition, KLF4 can transactivate C/EBP*β* by binding to the region of 1438-1134 KB upstream of the C/EBP*β* promoter and promote lipid differentiation [61]. KLF6 can form a complex with histone deacetylase-3 (HDAC3), inhibiting preadipocyte factor-1 (Pref-1) expression and promoting lipid differentiation [62]. KLF2 is highly expressed in adipose progenitors, and its expression decreases during the process of lipid differentiation. Overexpressed KLF2 can bind to the CACCC region of PPAR*γ*2 proximal promoter and inhibit lipid differentiation as well as the expression of PPAR*γ*, C/EBP*α*, and sterol-regulated element-binding proteins (SREBP) by inhibiting the promoter activity [63]. RNA sequence analysis showed that KLFl6 expression was decreased on the first day of adipocyte differentiation of 3T3-L1 cells. Adipocyte differentiation was promoted by KLF16 knockdown but was inhibited by KLF16 overexpression via inhibition of PPAR*γ* promoter activity [64]. In addition, KLF3 and KLF7 were also found to play a negative regulatory role in adipocyte differentiation [65, 66].

### 4.4. Signal Transducers and Activators of Transcription (STATs) and Adipocyte Differentiation

The activated STAT protein enters the nucleus as a dimer and binds to the target gene to regulate gene transcription. In the adipocyte differentiation of mouse 3T3-L1 cells, the expression of STAT1 and STAT5 was significantly increased, while that of STAT3 and STAT6 was not significantly changed [67]. In the adipocyte differentiation of human subcutaneous adipose precursor cells, STAT1 expression was significantly decreased [68], while the expression of STAT3 and STAT5 was increased and STAT6 expression was unchanged [69]. The role of STAT1 in adipocyte differentiation is not clear, because its expression trend in humans and mice differs during the adipocyte differentiation process. Early adipocyte differentiation of 3T3-L1 cells was inhibited by STAT1 agonist interferon *γ*. Loss of STAT1 in 3T3-L1 cells can rescue the inhibition of adipocyte differentiation caused by prostaglandin factor 2*α* [70]. Other studies have found that STAT1 is required for adipose differentiation, and STAT1 overexpression in C3H10T1/2 cells can prevent the inhibition of lipid differentiation caused by B-cell lymphoma-6 knockdown [71]. There was no abnormal adipose tissue in STAT1 knockout mice [72]. STAT3 not only affects the proliferation of 3T3-L1 cells but also coregulates their adipocyte differentiation with high mobility group protein 2 [73]. The FABP4 promoter was used to specifically knock out STAT3 in the adipose tissue of mice, and the results showed that mice weight significantly increased and the adipocyte quantity increased compared with the wild-type mice [74]. STAT5A and STAT5B have different effects on adipocyte differentiation. Abnormal adipose tissue was found in the mice with STAT5A or STAT5B knockout or double knockout, and the amount of adipose tissue was only one-fifth of the original adipose tissue in mice without knockdown [75].

### 4.5. Histone Modification in Adipocyte Differentiation

Histone deacetylase sirtuin (SIRT) 1 plays an important role in biological processes such as stress tolerance, energy metabolism, and cell differentiation [76]. During the adipocyte differentiation of C3H101/2 cells, SIRT1 expression decreased [77]. Overexpression of SIRT1 activated the Wnt signal, which caused the deacetylation of *β*-catenin. The accumulation of *β*-catenin in the nucleus could inhibit adipocyte differentiation. SIRT1 knockdown resulted in increased acetylation of the histones H3-K9 and H4-K16 in the secreted frizzled-related protein (sFRP) 1 and sFRP2 promoters, thereby promoting transcription of these genes and promoting lipid differentiation [78]. Forkhead box protein O (FOXO) 1 is a member of the transcription factor FOXO family. It can recruit cyclic AMP response element-binding protein (CBP)/histone acetyltransferase p300 to initiate an acetylation. The acetylated FOXO1 can be phosphorylated by phosphorylated protein kinase B (PKB/AKT). The phosphorylation of FOXO1 by AKT inhibits the transcriptional activation of FOXO1. The acetylation of FOXO1 lost the ability of DNA-binding affinity and promoted its shuttling from nuclei to cytoplasm [79]. SIRT1 and SIRT2 can deacetylate and active FOXO1. Activated FOXO1 (nonphosphorylated nuclear FOXO1) in the nucleus binds to the promoters of target genes encoding p21, p27, and PPAR*γ*, and initiates subsequent transcriptions [80]. SIRT2 inhibits the acetylation and phosphorylation of FOXO1, thereby induces the accumulation of activated FOXO1 in the nucleus. Activated FOXO1 could inhibit adipogenesis via PPAR*γ* [81–84]. Lysine-specific histone demethylase 1 (LSD1) expression increased during the adipocyte differentiation of 3T3-L1 cells. LSD1 could reduce the dimethylation levels of histone H3K9 and H3K4 in the C/EBP*α* promoter region, thereby promoting adipocyte differentiation [85]. SET domain-containing 8 (SETD8) catalyzed the monomethylation of H4K20 and promoted PPAR*γ* expression. The activation of PPAR*γ* transcriptional activity leads to the induction of monomethylated H4K20 and modification of PPAR*γ* and its targets, thereby promoting adipogenesis [86]. Enhancer of zeste homolog 2 (EZH2) is a methyltransferase and can bind methyl groups to histone H3K27, which is also necessary for lipid differentiation. The absence of EZH2 in brown fat precursors results in reduced levels of the Wnt promoter histone H3K27me3, which is also saved by the ectopic EZH2 expression or the use of a Wnt/*β*-catenin signal inhibitor [87]. In addition, histone demethylases such as lysine-specific histone demethylase (LSD/KDM) 4, KDM6, and histone lysine demethylase PHF2 are also involved in adipose differentiation, and KDM2B inhibits transcription factor activator protein 2*α* promoter via H3K4me3 and H3K36me2 [88].

## 5. Role of microRNA and Long Noncoding RNA in Adipogenesis

microRNA (miR) can bind and cut target genes or inhibit target gene translation. Endogenous siRNA can be produced by the action of Dicer enzyme and bind to a specific protein to change its cellular location [89]. Many kinds of miRs are involved in regulating adipocyte differentiation. The expression of miR-143 increased during the differentiation of adipose progenitor cells. Overexpression of miR-143 promoted gene expression involved in adipose differentiation and triglyceride accumulation. Inhibition of miR-143 prevented the adipose differentiation of human fat progenitor cells [90, 91]. Additionally, miR-8 promotes adipocyte differentiation by inhibiting Wnt signaling [92]. Moreover, miR-17-92, miR-103, miR-21, miR-519d, miR-210, miR-30, miR-204/211, and miR-375 also play a certain role in promoting adipocyte differentiation, while miR-130, miR-448, and let-7y inhibit lipid differentiation [93, 94]. In addition to miRs, long noncoding RNA (LncRNA) is a type of noncoding RNA and is important during epigenetic regulation and can form a double-stranded RNA complex with mRNA causes protein transcription. Lnc-u90926 inhibits adipocyte differentiation by inhibiting the transactivation of PPAR*γ*2 [95]. As a novel LncRNA, HOXA-AS3 expression increased during the adipose differentiation of MSCs, and HOXA-AS3 silencing reduced the marker gene of adipose differentiation and inhibited the adipose differentiation [96]. Zhu et al. [97] reported that HOXA-AS3 interacted with EZH2 to regulate lineage commitment of MSCs. HOXA -AS3 can regulate the trimethylation level of H3K27 in the Runx2 promoter region by binding to EZH2. Therefore, HOXA-AS3 is considered to be an epigenetic switch regulating MSCs lineage specificity [98]. Adipocyte differentiation-associated LncRNA can act as a competitive endogenous RNA of miR-204 in the process of lipid differentiation, thereby promoting the expression of SIRT1, the target gene of miR-204, and thus inhibiting lipid differentiation [99]. The LncRNA NEAT1 can also regulate adipocyte differentiation under the influence of miRNA140 [100]. Other LncRNA including LncRNA Blnc1 and Plnc 1 are also involved in regulating adipocyte differentiation [101, 102].

## 6. Other Biochemical Response Involved in Adipocyte Differentiation

### 6.1. Unfolded Protein Responses in Adipocyte Differentiation

**Figure 3:**
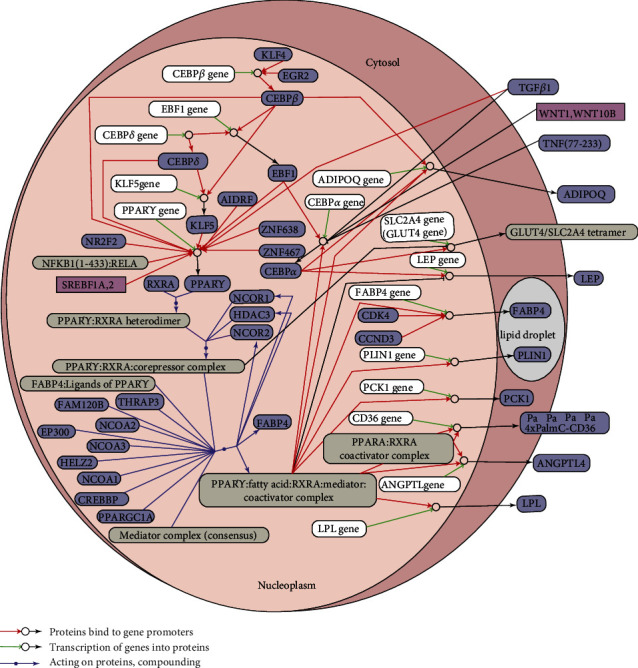
Regulation of adipocyte differentiation. A regulatory loop exists between PPAR*γ* and CEBP activation. Transcription factor Coe (EBF) activates CEBP*α*, CEBP*α* activates EBF1, and EBF1 activates PPAR*γ*. CEBP*β* and CEBP*δ* act directly on the PPAR*γ* gene by binding its promoter and activating transcription. CEBP*α*, CEBP*β*, and CEBP*δ* can activate the EBF1 gene and KLF5. The EBF1 and KLF5 proteins in turn bind the promoter of PPAR*γ*, which becomes activated. Other hormones, such as insulin, can affect the expression of PPAR*γ* and other transcription factors, such as SREBP1c. PPAR*γ* can form a heterodimer with the RXR*α*. In the absence of activating ligands, the PPAR*γ*-RXR*α* complex recruits transcription repressors, such as nuclear receptor corepressor (NCoR) 2, NCoR1, and HDAC3. Upon binding with activating ligands, PPAR*γ* causes a rearrangement of adjacent factors. Corepressors such as NCoR2 are lost, and coactivators such as Transcription intermediary factor TIF2, CBP, and p300 are recruited, which can result in the expression of Cyclic AMP-responsive element-binding protein (CREB) followed by PPAR*γ*. PPAR*γ* expression initiates the expression of downstream genes, including angiopoietin-related protein PGAR, Perilipin, FABP4, CEBP*α*, fatty acid transport-related proteins, carbohydrate metabolism-related proteins, and energy homeostasis-related proteins.

In the endoplasmic reticulum of eukaryotes, unfolded protein response involves three proteins: inositol-requiring enzyme 1*α*, double-stranded RNA-dependent protein kinase-like ER kinase, and activating transcription factor (ATF) 6*α* [103]. Knockdown of ATF6*α* affects the expression of adipocytes genes and inhibits C3H10T1/2 adipocyte differentiation [104]. The inhibitory effect of berberine on adipocyte differentiation of 3T3-L1 cells is also due to induced CHOP and decorin 2 expressions, and this inhibitory effect is ameliorated by CHOP knockout [105]. In the adipocyte differentiation process of 3T3-L1 cells, increases in PPAR*γ* and C/EBP*α* as markers of adipocyte differentiation were accompanied by an increase in the corresponding protein expressions of phosphorylated Eukaryotic translation initiation factor (EIF) 2*α*, phosphorylated endoribonuclease IRE1*α*, ATF4, CHOP, and other unfolded protein responses. Endoplasmic reticulum stress inducer or hypoxic endoplasmic reticulum stress can inhibit adipocyte differentiation. Additionally, EIF2*α* mutation results in continuous activation or overexpression of CHOP, which also inhibits adipocyte differentiation [106]. After the initiation of adipose differentiation, numerous differentiation-associated proteins are synthesized. Exogenous endoplasmic reticulum stress inducers can lead to excessive endoplasmic reticulum response, which in turn affects the synthesis of proteins related to differentiation and inhibits adipocyte formation (Figure 3).

### 6.2. Role of Oxidative Stress in Adipogenesis

During the directional differentiation of MSCs, mitochondrial complex I and III, and NADPH oxidase NOX4 are the main sources of oxygen species (ROS) production. Currently, it is believed that ROS affects not only the cell cycle and apoptosis but also differentiation through influencing the signaling pathways including the Wnt, HH, and FOXO signaling cascade during MSCs differentiation [107]. The differentiation ability of stem cells is determined by the arrangement of perinuclear mitochondria, which specifically manifests as low ATP/cell contents and a high rate of oxygen consumption. The lack of these characteristics indicates stem cell differentiation [108]. Adipocyte differentiation is a highly dependent ROS activation factor related to mitosis and cell maturation [109]. Schroder et al. found that exogenous H_2_O_2_ could stimulate adipocyte differentiation of mouse 3T3-L1 cells and human adipocyte progenitor cells in the absence of insulin. H_2_O_2_ regulates adipocyte differentiation of 3T3-L1 cells in a dose-dependent manner. High doses of H_2_O_2_ (1, 10, and 30 *μ*M) promote adipocyte differentiation [110, 111]. Tormos et al. found that ROS synthesis increased in human MSCs at the early stage of adipose differentiation, and targeted antioxidants could inhibit lipid differentiation. By knocking down Rieske iron-sulfur protein and ubiquinone-binding protein, ROS produced by mitochondrial complex III was found to be necessary in initiating adipose differentiation [112]. However, other studies have shown that the expression levels of adiponectin and PPAR*γ* were decreased by using H_2_O_2_ (0.1–0.5 mM) in 3T3-L1 cells [113]. Free radical nitric oxide (NO) also promotes lipid differentiation, because treatment with NO inducer hydroxylamine or NO synthase (NOS) substrate arginine can significantly induce adipose differentiation of rat adipose progenitor cells. NOS induced adipose differentiation mainly via eNOS rather than iNOS [114]. ROS can induce adipose differentiation primarily by inhibiting Wnt, FOXO, and HH signaling pathways that inhibit lipid differentiation.

### 6.3. Autophagy in Adipocyte Differentiation

The increase in autophagosomes during lipid differentiation indicates that autophagy may play an important role in lipid differentiation [115]. Baerga et al. confirmed that the adipocyte differentiation efficiency was significantly inhibited in mouse embryonic fibroblasts lacking autophagy-related gene (Atg) 5, a gene encoding an essential protein required for autophagy [116]. Knockdown of Atg5 in 3T3-L1 cells promotes proteasome-dependent degradation of PPAR*γ*2, thereby inhibiting adipocyte differentiation [117]. Zhang reported that autophagy-related gene 7(Atg7) is also crucial for adipose development. Atg7-deficient mice were slim and only had 20% of white fat compared to wild-type mice, and the lipid metabolism and hormone-induced lipolysis in the adipocytes were altered [118]. Autophagy related gene Atg4b is activated by C/EBP*β* in the process of lipid differentiation, and autophagy activation is necessary for the degradation of Klf2 and Klf3, two negative regulators of lipid differentiation. These results showed that adipose differentiation and autophagy are mutually complementary [119]. In 3T3-L1 cells, autophagy was inhibited by aspartate ammonia or 3-methyladenine at different lipid induction periods (0–2, 2–4, 4–6, and 6–8 days), and only autophagy inhibition at 0–2 days hindered the formation of lipid droplets and the expression of lipid marker genes, indicating that autophagy was very important in the early stage of lipid differentiation [120]. Recent studies showed that LC3 is overexpressed in 3T3-L1 cells, further demonstrating the important role of autophagy in lipid differentiation [121].

### 6.4. Role of Alternative Splicing in Adipogenesis

Selective splicing is influenced by splicing regulators, which regulate adipocyte differentiation by regulating the selective splicing of genes specific to this process. Lipin1 is an important regulator in the process of adipocyte differentiation and includes two isomers, Lipin1*α* and Lipin1*β*, which have different effects. High expression of Lipin1*α* promotes adipocyte differentiation, while that of Lipin1*β* promotes lipid droplet formation [122]. In Sam68-deficient mice, the fifth intron of serine/threonine-protein kinase mTOR was retained, resulting in unstable and rapid mTOR degradation and inhibition of adipocyte differentiation [123]. Furthermore, there are four isomers of Pref-1, Pref-1a and Pref-1b can inhibit adipocyte differentiation of 3T3-L1, while Pref-1c and Pref-1d have no effect on this process [124].

### 6.5. Cytoskeletal Remodeling in Adipocyte Differentiation

During adipocyte differentiation from stem cells, morphological changes to cells due to remodeling of the actin cytoskeleton are the hallmark of differentiation. McBeath et al. showed that cell shape was associated with differentiation of human MSCs to adipocytes or osteoblasts. Flattened and spread cells underwent osteogenesis, while unspread, round cells became adipocytes. They demonstrated that mesenchymal cells mainly from mesoderm cells were more prone to adipocyte differentiation, while pinacocytes were more prone to osteogenic differentiation. Disruption of actin by cytochalasin D can significantly promote adipocyte differentiation [125]. The increase in the monomer G-actin interacts with megakaryoblastic leukemia 1 and inhibits its nuclear translocation, thereby promoting PPAR*γ* expression during adipocyte differentiation [126], while the mTORC2 signal and RhoA-ROCK mediate cytoskeletal remodeling and MSCs lineage selection [127]. During adipocyte differentiation, the formation of cortical actin structures starts with the accumulation of filamentous actin near the cell membrane. The cortical assembly and nucleation of actin are controlled by the actin-related protein 2/3 (Arp2/3) complex. Yang et al. found that Arp2/3 knockdown seriously inhibited adipocyte differentiation of cells, and the cortical actin cytoskeleton was very important for the secretion of GLUT4 particles into cells as well as insulin signal transduction [128].

## 7. Adipose Differentiation Induction of Malignant Tumor Cells

Tumors are considered to be heterogeneous ecosystems composed of a variety of tumor cell subsets and stromal cells. Cancer cells are typically characterized by uncontrolled proliferation and disorders of differentiation. The long-term self-renewal, proliferation capacity, and differentiation potential of CSCs are considered to be the major determinants of tumor recurrence, treatment failure and metastasis, and chemotherapy-resistant [129]. Only one subset of tumor cells can drive tumorigenesis and initiate the formation of heterogeneous tumors. It is important to note that any cell in the tumor may gain or lose its initiation-ability due to tumor microenvironment or therapeutic interventions. Therefore, CSCs should be considered as a state cell rather than a static subpopulation of cancer cells [130, 131]. Adipose differentiation of human MSCs could be induced by using a complex stimulus which includes dexamethasone, 3-isobutyl-1-methylxanthine, indomethacin, and insulin (a classical cocktail) [132]. Our previous study has shown that polyploid giant cancer cells (PGCCs) had the properties of CSCs and can be induced into adipose *in vitro* and *in vivo* [133].

### 7.1. Malignant Tumor Cells Can Be Induced Adipocytes

WDLPS and dedifferentiated DDLPS are the most common types of liposarcoma. WDLPS/DDLPS cells can be induced to differentiate into adipocytes by dexamethasone, indomethacin, insulin, and IBMX. In vitro experiments have shown that these four compounds induce adipogenesis by upregulation of transcription and translation of genes involved in maintaining cancer cell stemness and adipogenic differentiation, which might be used in the clinical treatment of DDLPS patients in the future [10]. In vivo, the induction of adipogenesis inhibited the tumorigenic ability of DDLPS. The tumor suppressor protein *p*53 is the negative regulator of adipocyte formation and the positive regulator of insulin sensitivity [134]. In theory, adipocyte-inducing agent can result in the least partial differentiation of tumor cells, reducing their malignant phenotype in *p*53 deficient tumors. The adipogenic differentiation potential is promising in the treatment of cancer cell-derived from *p*53 deletion fibroblast.

### 7.2. Adipocyte Differentiation of CSCs

CSCs are a very small population of cancer cells that exist in tumor tissue and closely related to the occurrence, development, metastasis, recurrence, and drug resistance of malignant tumors [135]. Adipocytes can derive not only from preadipocytes and pluripotent MSCs but also CSCs. Our previous study has shown that cobalt chloride (CoCl_2_) was used to treat different cancer cell lines and daughter cells derived from PGCCs gained a mesenchymal phenotype [133]. When cultured with adipogenesis medium, PGCCs can differentiate into adipocytes [133].

### 7.3. Molecular Mechanism of CSCs Differentiating into Adipose

The molecular mechanism of adipose differentiation of CSCs is similar to that of MSCs. PPAR*γ* activation is the key to adipocyte differentiation of CSCs in vivo. The phosphorylation of FOXO1 by AKT inhibited the transcriptional activation of FOXO1 and activated FOXO1 could inhibit adipogenesis via PPAR*γ* [80]. Activated AKT1 after phosphorylation was of great significance to promote adipogenesis via mTORC2-AKT1-FOXC2 signal pathway [136]. PI3K/AKT plays an important role in maintaining the stemness of various CSCs. The expression of OCT4 and Nanog in breast CSCs depended on the PI3K/AKT pathway [137]. Cytochrome c oxidase 2 inducing the formation of CSCs in breast cancer was involved in the activation of PI3K and AKT [138]. Activating PI3K/AKT signal pathway promoted the initiation of liver CSCs [139]. Inhibition of PI3K/Akt/mTOR pathway suppressed the stemness of colon CSCs [140]. AKT signal pathway plays an important role both in the formation and differentiation of CSCs. The nuclear oncoprotein Myc was a pivotal regulator in cell cycle regulation, proliferation, differentiation, and apoptosis [141, 142]. Deregulated Myc expression was incompatible with terminal differentiation in a variety of cell types, including adipocytes [141].

PARP is a DNA repair enzyme and plays an important role in DNA damage repair and apoptosis. PARP family members are associated with CSC biology and its inhibition, including the development, neurogenesis, and adipogenesis of stem cells. PARP1 and PARP2 are crucial for adipocyte differentiation and the regulation of lipid accumulation [143]. PARP1 can keep the preadipocytes in the stationary phase of growth and inhibits the formation of adipocytes [144]. PARP-1 can mediate poly-ADP-ribosylation (PARylation) of CEBP*β* and PARylation is a posttranslational modification of proteins mediated by PARP family members. CEBP*β* is the crucial transcription factor in adipogenesis. The PARylation of CEBP*β* changes its DNA binding and transcriptional activities and thus inhibits the adipocyte differentiation of stem cells. Depletion or chemical inhibition of PARP-1, or mutation of the PARylation sites on C/EBP*β*, promotes early adipogenesis [144]. PPAR*γ* binding with RXR forms different heterodimers plays a central role in white adipose tissue (WAT) differentiation and function, regulating the expression of key WAT proteins [145]. PARP-2 is a member of the PPAR*γ*/RXR transcription machinery and a novel cofactor of PPAR activity. PARP-2 overexpression enhanced the basal activity of PPAR*γ* and PARP-2(-/-) mouse embryonic fibroblasts failed to differentiate into adipocytes. In transient transfection assays, PARP-2 siRNA decreases basal activity and ligand-dependent activation of PPAR*γ*. Chromatin immunoprecipitation has shown a DNA-dependent interaction of PARP-2 and PPAR*γ*/RXR heterodimer [145].

### 7.4. Differentiation Therapy of Malignant Tumor

CSC-specific phenotypes and mechanisms indicate that CSCs may contribute to the failure of existing therapies to consistently eradicate malignant tumors [146]. Differentiation of primitive cells within a malignancy may lead to tumor degeneration and increased susceptibility to conventional cytotoxic anticancer therapies [147]. Differentiation therapy has been recognized for a long time, and potential strategies are that induce quiescent CSCs to differentiate into more mature tumor cells. Results of Piccirillo et al. showed that BMP4 induced glioblastoma differentiation in mice models of human glioblastoma [148]. Modulation of CSC signaling pathways has also shown the differentiation of CSCs in medulloblastoma [149]. In human breast cancer, Gupta et al. identified that potassium ionophore and salinomycin could induce epithelial differentiation of tumor cells and result in inhibition of tumor growth [150]. In addition, the effects of the inhibitor and agonist for SIRT1/2 on the inducing osteogenic differentiation indicated that SIRT1/2 had an important role in this process. Inducing differentiation of cancer cells may have potentially translational applications in the treatment of SCLC [6]. EMT plays a critical role in tumor formation and differentiation. EMT is involved in tumor metastasis and is highly correlated with tumor progression. Cancer cells undergo EMT to exhibit a high degree of plasticity, which many studies have begun to exploit therapeutically by forcing the transdifferentiation of EMT-derived cancer cells into benign cells. Ishay-Ronen et al. showed that plasticity intrinsic to the EMT program could be exploited to divert cancer cells into becoming postmitotic adipocytes, thus preventing the metastases of cancer. A cocktail of rosiglitazone and BMP2 (a member of the transforming growth factor *β* [TGF-*β*] superfamily) was shown to influence the cells with mesenchymal phenotype, but not epithelial phenotype [3]. The key step of adipocyte differentiation in tumors is the same as that in MSCs with activation of the transcription factor PPAR*γ*. The study refers to stem cells derived from the breast cancer microenvironment can be induced differentiation by and their impaired adipogenesis. The PPAR*γ* agonist thiazolidinedione delays the invasive progression and induces adipose differentiation of ductal carcinoma in situ [3]. Adipose-derived stem cells could be induced into adipocytes by the PPAR*γ* agonist thiazolidinedione, which was impaired by breast cancer microenvironment [151]. The adipocytes derived from breast cancer cells are truly functional adipocytes. They express adipocyte-specific markers (such as C/EPB genes, PPAR*γ*2, FABP4) that show similar adipocyte metabolic and transcriptome characteristics of adipocytes and lack mesenchymal morphological features. The induced cells strongly expressed CEBP*α* and formed lipid droplets. Two FDA-approved drugs were used to treat animal xenografts from breast cancer cells. The two drugs are a combination of rosiglitazone, a PPAR inhibitor widely used to treat diabetes, and trametinib, a MEK inhibitor. Compared with the treatment drug trametinib alone, the combination treatment did not significantly inhibit tumor growth, but it significantly inhibited tumor invasion and metastasis. The combination treatment had no toxic effect on mice. Preclinical models further confirmed the effectiveness of this adipogenesis therapy. A high number of human adipocytes were detected in primary tumors treated with the combination of rosiglitazone and trametinib, and a significant decrease was found in tumor cells metastasized to the lung [3].

PARP inhibition can induce the transdifferentiation of white adipocytes to brown-like adipocytes, and the activity of PARP may be a determinant of the differentiation of these adipocyte lineages [152]. Olaparib, a potent PARP inhibitor used in clinical, can induce white adipocytes to transdifferentiate into brown/beige adipocytes with smaller lipid droplets. Olaparib can inhibit nuclear and cytosolic poly-ADP-ribose formation, induced NAD+/NADH ratio, and consequently enhanced SIRT1 and AMPK activity [152]. PARP inhibitors enhance the cytotoxic effects of antitumor drugs and radiotherapy and selectively kill tumor cells with homologous recombination deficiency, such as BRCA1 or BRCA2 mutations [153, 154]. Olaparib is the first small molecule PARP inhibitor compound approved by the FDA and EMA to enter the clinic in 2014 for the treatment of advanced-stage BRCA1/2-mutated ovarian cancers. In a phase III clinical trial of pancreatic cancer patients in 2019, Olaparib achieved positive results in progression-free survival [154, 155]. Rucaparib is an inhibitor of PARP, and it disrupts DNA repair and replication pathways, leading to the selective killing of cancer cells with BRCA1/2 mutations [144]. In addition, the expression of S100A16 in human breast cancer tissues was higher than in the paired adjacent noncancerous tissues. S100A16 is a calcium-binding signaling protein, promotes adipogenesis, and involved in weight gain attenuation induced by dietary calcium. Enhanced adipogenesis with more lipid droplet density was clearly observed in 3T3-L1 preadipocytes with overexpression of S100A16 [156]. S100A16 promoted EMT by upregulating the transcription factors Notch1, ZEB1, and ZEB2, which had the capacities to directly repress the expression of epithelial markers E-cadherin and beta-catenin but increase mesenchymal markers N-cadherin and vimentin [5]. All the results regarding differentiation therapy may hold great promise for new therapeutic strategies. Many targeted differentiation therapies for CSCs are currently undergoing preclinical and clinical research with the aim of reducing tumor recurrence and metastatic spread.

## 8. Conclusions

Adipocyte differentiation is a complex process, and a series of molecular and signaling pathways have been identified as involved in regulating adipocyte differentiation. MSCs, which are recruited from the vascular stroma of adipose tissue, provide the adipocyte precursors. Members of the BMP and Wnt families are key mediators of stem cell commitment to produce preadipocytes. In addition, exposure of growth-arrested preadipocytes to differentiation inducers such as IGF1, glucocorticoid, and cAMP triggers DNA replication and reentry into mitotic clonal expansion, which involves a transcription factor cascade followed by the expression of adipocyte genes. Critical to these events are phosphorylation of the transcription factor C/EBP*β* by MAP kinase and GSK3*β*, and activated C/EBP*β* then triggers transcription of PPAR*γ* and C/EBP*α*, which in turn coordinately activate genes whose expression produces the adipocyte phenotype.

CSCs play an important fundamental role in tumor progression because of their tumorigenic properties, resistance to radiation and chemotherapy, invasiveness, and tendency to evade immune responses, which contribute to tumor recurrence. The difficulty of targeting CSCs lies in the intrinsic properties of these cells and the acquired phenotypes following therapeutic interventions. These characteristics underscore the importance of innovative treatment options. Cell differentiation is an important pathway in tumor transformation, and a better understanding of typical differentiation factors may open the door to new therapeutic strategies that regulate key differentiation pathways in cancer. Although the understanding of the process of adipocyte differentiation has improved over the past 20 years, many questions remain. For example, how can adipocyte differentiation be induced in vivo? How can the different differentiation directions of pluripotent stem cells be balanced? Can we avoid diseases by influencing the adipocyte differentiation of stem cells? Almost every important cellular signaling pathway has a positive or negative effect on adipocyte development, and some pathways exert both pro- and antiadipogenic effects depending on factors that are still poorly understood. Many targeted differentiation therapies for CSCs are currently undergoing preclinical and clinical research with the aim of reducing recurrence and metastatic spread. Current and future studies will provide strong evidence for solving various problems and focus on accurate targets for the treatment of adipocyte differentiation-related diseases. Studies about the molecular mechanism and regulatory proteins involved in adipocyte differentiation of MSCs may provide new therapeutic ideas and targets for clinical malignant tumor differentiation therapy.

## Abbreviations

MSCs: Mesenchymal stem cells
CSCs: Cancer stem cells
PGCCs: Polyploid giant cancer cells
IBMX: 3-isobutyl-1-methylxanthine
EMT: Epithelial-to-mesenchymal transition
BRCA: Breast cancer sususceptibility gene
WDLPS: Well-differentiated liposarcoma
DDLPS: Dedifferentiated liposarcoma
BMP: Bone morphogenetic protein
C/EBPs: CCAAT enhancer-binding proteins
CHOP: C/EBP-homologous protein
CBP: Cyclic AMP response element-binding protein
FABP4: Fatty acid-binding protein 4
PPAR*γ*: Peroxisome proliferator-activated receptor *γ*
PPRE: Peroxisome proliferator response element
PPFP: PPAR*γ* fusion protein
PARP: Poly ADP-ribose polymerase
RXR: Retinoid X receptor
IGF1: Insulin/insulin-like growth factor 1
TGF: Transforming growth factor
TNF: Tumor necrosis factor
FGF: Fibroblast growth factor
MMP: Matrix metalloproteinase
SMAD: Drosophila mothers against decapentaplegic protein
ERK: Extracellular regulated protein kinases
MAPK: Mitogen-activated protein kinase
MKP1: Mitogen-activated protein kinase phosphatase-1
JAK-STAT3: Janus kinase-signal transducer and activator of transcription 3
PI3K: Phosphoinositide 3-kinase
AKT/PKB: Protein kinase B
mTOR: Serine/threonine-protein kinase
KLF: Krüppel-like factors
HH: Hedgehog
HDAC: Histone deacetylase
SREBP: Sterol-regulatory element-binding proteins
Pref: Preadipocyte factor
GLUT4: Glucose transporter 4
LSD1/KDM1: Lysine specific demethylase 1 A
SIRT1: sirtuin1
sFRP: Secreted frizzled-related protein
FOXO: Forkhead box protein O
P300: Histone acetyltransferase p300
SETD: SET domain-containing
HOX: Homeotic genes
LncRNA: Long noncoding RNA
Arp2/3: Actin-related protein 2/3
PARylation: Poly-ADP-ribosylation
EIF: Eukaryotic translation initiation factor
ROS: Oxygen species
Atg: Autophagy-related gene
EBF: Transcription factor Coe
NCoR: Nuclear receptor corepressor.
